# Discovery of a Novel Na_v_1.7 Inhibitor From *Cyriopagopus albostriatus* Venom With Potent Analgesic Efficacy

**DOI:** 10.3389/fphar.2018.01158

**Published:** 2018-10-16

**Authors:** Yunxiao Zhang, Dezheng Peng, Biao Huang, Qiuchu Yang, Qingfeng Zhang, Minzhi Chen, Mingqiang Rong, Zhonghua Liu

**Affiliations:** The National and Local Joint Engineering Laboratory of Animal Peptide Drug Development, College of Life Sciences, Hunan Normal University, Changsha, China

**Keywords:** sodium channel, electrophysiology, tarantula spider, peptide toxin, Na_v_1.7, analgesic activity

## Abstract

Spider venoms contain a vast array of bioactive peptides targeting ion channels. A large number of peptides have high potency and selectivity toward sodium channels. Na_v_1.7 contributes to action potential generation and propagation and participates in pain signaling pathway. In this study, we describe the identification of μ-TRTX-Ca2a (Ca2a), a novel 35-residue peptide from the venom of Vietnam spider *Cyriopagopus albostriatus* (*C. albostriatus*) that potently inhibits Na_v_1.7 (IC_50_ = 98.1 ± 3.3 nM) with high selectivity against skeletal muscle isoform Na_v_1.4 (IC_50_ > 10 μM) and cardiac muscle isoform Na_v_1.5 (IC_50_ > 10 μM). Ca2a did not significantly alter the voltage-dependent activation or fast inactivation of Na_v_1.7, but it hyperpolarized the slow inactivation. Site-directed mutagenesis analysis indicated that Ca2a bound with Na_v_1.7 at the extracellular S3–S4 linker of domain II. Meanwhile, Ca2a dose-dependently attenuated pain behaviors in rodent models of formalin-induced paw licking, hot plate test, and acetic acid-induced writhing. This study indicates that Ca2a is a potential lead molecule for drug development of novel analgesics.

## Introduction

Voltage-gated sodium channels (VGSCs) are important integral membrane proteins expressed in electrically excitable cells. The opening of pore-forming α subunits causes an influx of sodium ions, which is essential for action potential generation and propagation. VGSCs are composed of α subunits in association with one or more auxiliary β subunits (Catterall, 2000, 2012, 2014). The α subunits are organized in four homologous domains (DI–DIV), each of which consists of six transmembrane α helices (S1–S6) connected by extracellular and intracellular loops. Up to now, nine distinct VGSC α subunits (Na_v_1.1–1.9) and four β subunits have been cloned from mammals (Dib-Hajj et al., 2010). Compelling genetic studies and clinical evidence have revealed the importance of human Na_v_1.7 (hNa_v_1.7) as an analgesic target (Dib-Hajj et al., 2009; Wang et al., 2011; Gingras et al., 2014).

Loss-of-function mutations in *SCN9A*, the gene encoding hNa_v_1.7, have been identified as a cause of congenital insensitivity to pain (CIP) (Mansouri et al., 2014; Shorer et al., 2014), while gain-of-function mutations of *SCN9A* are the cause of several pain disorders, including inherited erythromelalgia (IEM) (Wu et al., 2017), paroxysmal extreme pain disorder (PEPD) (Fertleman et al., 2006) and small fiber neuropathy (Faber et al., 2012). Therefore, chemicals pharmacologically inhibiting hNa_v_1.7 activity have the potential to treat chronic pain. Developing analgesics against hNa_v_1.7 with Na_v_ subtype selectivity is essential, because α subunit shares high sequence similarity between each other, and off-target may cause serious side effects, especially Na_v_1.4 expressed in skeletal muscle and Na_v_1.5 expressed in cardiac muscle.

Spider venom is a highly complex mixture, mainly containing protein, polypeptide, and small molecules. Polypeptide toxins can specifically interact with ion channel proteins, membrane receptors, and transporters, and the spider venom-derived peptide toxins were used as a potential rich source of drug discovery (Escoubas and King, 2009; King, 2011). Most venom peptides have disulfide-rich architectures that provide extreme stability and a high level of resistance to proteases, which are necessary characteristics for drug discovery and design. The venom of spider *Cyriopagopus albostriatus* (C. *albostriatus*) has not been well investigated yet. Here we reported the isolation and characterization of μ-TRTX-Ca2a (Ca2a), a 35-residue peptide isolated from the venom of Vietnam Tarantula C. *albostriatus* with high potency and selectivity against Na_v_1.7. Rodent pain models demonstrated that Ca2a had powerful analgesic effects.

## Materials and Methods

### Purification of Peptide

The crude venom of *C. albostriatus* was obtained by electronic stimulation, and preserved at −80°C after lyophilization. The lyophilized venom was dissolved in ddH_2_O to a final concentration of 5 mg/ml and subjected to the first round of semi-preparative RP-HPLC purification (C18 column, 10 mm × 250 mm, 5 μm, Welch, Shanghai, China) using linear acetonitrile gradient ranging from 10 to 55% with an increasing rate of 1% per minute (Waters e2695 Separations Module, Waters, CA, United States). The fraction containing Ca2a was then collected, lyophilized, and subjected to a second round of analytical RP-HPLC purification (C18 column, 4.6 mm × 250 mm, 5 μm, Welch, Shanghai, China). The acetonitrile gradient was increased ranging from 20 to 40% at an increasing rate of 1% per minute (Waters 2795 Separations Module, Waters, CA, United States). Fractions were lyophilized and stored at −20°C before use. The purity of the toxin was tested by MALDI-TOF MS analysis (AB SCIEX TOF/TOF^TM^ 5800 system, Applied Biosystems, United States).

### Plasmid and Transfection

The cDNA genes encoding rat Na_v_1.4 and human Na_v_1.7 were subcloned into vectors pRGB4 and pcDNA3.1-mod, respectively. Mutations of rNa_v_1.4 (N655D, Q657E, and N655D/Q657E) and hNa_v_1.7 (D816N, E818Q, and D816N/E818Q) were constructed using the Gene Tailor Site-Directed Mutagenesis system (Invitrogen, Carlsbad, CA, United States), according to the manufacturer’s instructions. Na_v_1.2–Na_v_1.7 and mutant plasmids together with eGFP were transiently transfected into HEK293T cells while Na_v_1.8 together with eGFP was transiently transfected into ND7/23 cells by Lipofectamine 2000 (Invitrogen, Carlsbad, CA, United States). Additionally, plasmids β1- and β2-eGFP encoding the human β1 and β2 subunits, respectively, were co-transfected with those encoding WT Na_v_1.7 and Na_v_1.7 mutations in HEK293T cells. Human Na_v_1.9 was transfected into ND7/23 cells according to a previous report (Zhou et al., 2017). HEK293T and ND7/23 cells were grown under standard tissue culture conditions (5% CO_2_, 37°C) in Dulbecco’s modified Eagle’s medium (DMEM) supplemented with 10% fetal bovine serum (FBS). Cells with green fluorescent protein fluorescence were selected for whole-cell patch-clamp recordings 24 h after transfection.

### Whole-Cell Patch-Clamp Recordings

Whole-cell patch-clamp recordings were performed at room temperature (20–25°C) using an EPC 10 USB Patch Clamp Amplifier (HEKA, Elektronik, Lambrecht, Germany). Suction pipettes with access resistance of 2.0–3.0 MΩ were made from borosilicate glass capillary tubes (thickness = 0.225 mm) using a two-step vertical microelectrode puller (PC-10; Narishige, Tokyo, Japan). The standard pipet solution contained (in mM): 140 CsCl, 10 NaCl, 1 EGTA, and 10 HEPES (pH 7.4). Bath solution contained (in mM): 140 NaCl, 2 CaCl_2_, 1 MgCl_2_, 5 KCl, 20 HEPES (pH 7.4), and 10 glucose. All chemicals were the products of Sigma-Aldrich (St. Louis, MO, United States) and dissolved in water. Data was acquired by PatchMaster software (HEKA Elektronik, Lambrecht, Germany). Data was analyzed by software Igo Pro 6.10A (WaveMetrics, Lake Oswego, OR, United States), SigmaPlot 10.0 (Sigma-Aldrich, St. Louis, MO, United States), OriginPro 8 (OriginLab Corp., Northampton, MA, United States), and GraphPad Prism 5 (GraphPad Software, San Diego, CA, United States). Membrane currents were filtered at 5 kHz and sampled at 20 kHz. To minimize voltage errors, 80–90% series resistance compensation was applied. Voltage-clamp recordings were acquired 5 min after establishing whole-cell configuration to allow adequate equilibration between the micropipette solution and the cell interior.

The Na_v_1.2–Na_v_1.7 channel currents were elicited by 50 ms depolarization potential to −10 mV from the holding voltage of −100 mV. The depolarization potential for Na_v_1.8 was +20 mV. The Na_v_1.9 current was elicited by 50 ms depolarization potential to −40 mV from the holding voltage of −120 mV in the presence of 1 μM TTX.

To measure current–voltage (I–V) relationships, a range of potentials from −100 mV to +80 mV in 5 mV increments were applied from the holding potential (−100 mV) for 50 ms at 5 s intervals. Peak values at each potential were plotted to form I–V curves. Activation curves were obtained by calculating the conductance G at each voltage. G = I/(V − V _rev_), with _V _rev__ being the reversal potential, determined for each cell individually. Steady-state fast inactivation was assessed with a 20-ms depolarizing test potential of −10 mV following a 500-ms prepulse at potentials that ranged from −110 to −30 mV with a 10-mV increment.

Fast inactivation time constants were calculated by fitting current decay traces with a single exponential function using the I–V protocol described above. Recovery from fast-inactivation (repriming) was assessed by using a two-pulse protocol consisting of a depolarizing pulse to −10 mV for 50 ms to inactivate channels, followed by a step to −100 mV of variable duration (1 to 1024 ms) to promote recovery, and 50 ms test pulse to −10 mV to assess availability of channels. Voltage dependence of steady-state slow inactivation was measured using a series of 15 s pre-pulses, ranging from −120 to 0 mV in 10-mV increments, followed by a 50 ms step to −100 mV to remove fast inactivation, and a 50 ms test pulse to −10 mV to assess the available non-inactivated channels. The rate of toxin dissociation was determined by stepping to a depolarizing pulse of 100, 80, or 60 mV for various durations followed by a 500 ms hyperpolarization to −100 mV to allow recovery from fast inactivation, and then assessing the effect of the depolarizing pulse with a 50-ms test pulse to −10 mV. Very little re-binding takes place due to slow kinetics of the blocking of the channel during 500-ms hyperpolarization to −100 mV. Use/frequency-dependent inhibition of the channel was measured by applying repetitive pulses of different frequencies (1, 5, and 10 Hz) that mimic high firing frequency of DRG neurons expressing Na_v_1.7.

### Animals

The ICR mice (18–22 g) used in this study were purchased from the Experimental Animal Center of SLac-kinda (Changsha, China). The animals were maintained at 20–25°C and freely allowed to standard rodent chow and water *ad libitum*. Ethical approval for *in vivo* experiments in animals was approved by the Animal Care and Use Committee (ACUC) at the Hunan Province Animal Management Office (HPAMO).

### Formalin-Induced Paw Licking

A formalin test was performed according to the previous method (Owoyele et al., 2005). Mice were intraperitoneally injected with saline, morphine or Ca2a 30 min before injection with 20 μL formalin (5%) solution under the plantar surface of the right hind paw. The time spent licking the injected paw by each mouse was recorded by a digital stopwatch during Phase I (0–15 min post-injection) and Phase II (15–40 min post-injection).

### Hot Plate Test

According to a previous method (Meng et al., 2017), a hot plate apparatus (model YLS-21A, Jinan, China) maintained at 55 ± 1°C, was used to measure the pain threshold of mice subjected to a thermal stimulus. Each female mouse was placed on hot plate to observe its pain response (hind-paw-licking or jumping) acted as its own control. The mice whose latent response times were shorter than 5 s or longer than 30 s were excluded from the test. The saline, morphine, and Ca2a were intraperitoneally injected to mice and the latent response time was recorded at 0.5, 1, 1.5, and 2 h.

### Abdominal Writhing Induced by Acetic Acid

According to the method previously described (Liu et al., 2014), mice were injected intraperitoneally with a saline, morphine, or Ca2a for 15 min prior to injection with 200 μL of 0.8% (*v/v*) acetic acid solution, which induced abdominal contraction and hind limb stretching. The abdominal writhing responses were counted for 30 min continuously.

### Data Analysis

Concentration-response curves were fitted using the following Hill logistic equation: *y* = *f*_max_ − (*f*_max_ − *f*_min_)/(1 + (*x*/*IC*_50_)^*n*^), where *f_max_* and *f*_*min*,_ respectively, represent the channel’s maximum and minimum responses to toxins, *f_min_* was set to 0, *x* represents toxin concentration and *n* is an empirical Hill coefficient. Activation curves were fitted with the Boltzmann equation: *y* = 1/(1 + exp(*V − V*_1/2_/*k*)), in which *V* is the test potential, *V*_1/2_ is the midpoint voltage of kinetics, and *k* is the slope factor. Peak inward currents from steady-state inactivation were normalized by the maximum current amplitude and fit with a Boltzmann equation *I* = *I*_min_ + 1/(1 + exp[*V*_m_ − *V*_1/2_/*k*]) where *I* is the current amplitude measured during the test depolarization, *V*_1/2_ is the midpoint of inaction, and *k* is the slope factor. The currents recovery from inactivation were fitted using a single exponential equation *f*(*t*) = *Ae^−t/T^ + C*, where *A* represents the amplitude of the current, *t* is the time, τ is the time constant, and *C* is the steady-state asymptote. Statistical analyses were performed using paired student’s *t*-test or ANOVA with paired comparisons. Results with *p* < 0.05 were considered significant. All data are presented as mean ± SEM.

## Results

### Isolation of Ca2a From *C. albostriatus*

**FIGURE 1:**
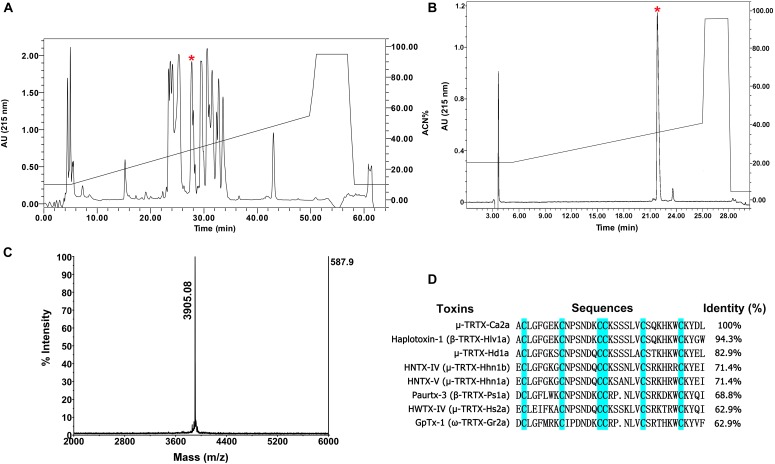
Identification of μ-TRTX-Ca2a from the venom of spider *C. albostriatus.* **(A)** Isolation of native Ca2a from the pooled protein fraction by a C18 RP-HPLC column. **(B)** Ca2a was purified to homogeneity by analytical RP-HPLC. **(C)** MALDI-TOF MS analysis showing a single predominant mass of 3905.08 Da. **(D)** Cysteines shaded in *cyan* formed disulfide bonds. Sequence alignment of Ca2a with related toxins. N-terminal sequencing analysis revealed a peptide with 35 residues containing 6 cysteines.

The venom of spider *C. albostriatus* was purified by C18 RP-HPLC. The eluted fractions were lyophilized and tested on pain-related ion channel hNa_v_1.7 heterologously expressed in HEK293T cells. The peak labeled with the red asterisk showed inhibition activity against Na_v_1.7 (**Figure 1A**). This fraction was further purified by analytical RP-HPLC (**Figure 1B**, asterisk labeled peak). MALDI-TOF MS analysis revealed that this peak represented a peptide toxin with a molecular weight of 3905.08 Da (**Figure 1C**). N-terminal Edman sequencing disclosed that the novel peptide toxin contained 35 amino acid residues (**Figure 1D**) and was named μ-TRTX-Ca2a (Ca2a). Ca2a contained six cysteines and belonged to the Family 1 of NaSpTx conforming to a conserved cysteine pattern of ICK motif. This family is comprised by spider venom-derived Na_v_ channel toxins with 33–35 residues forming a hyper-stable ICK motif (Klint et al., 2012).

### Selectivity of Ca2a for Sodium Channel Subtypes

**FIGURE 2:**
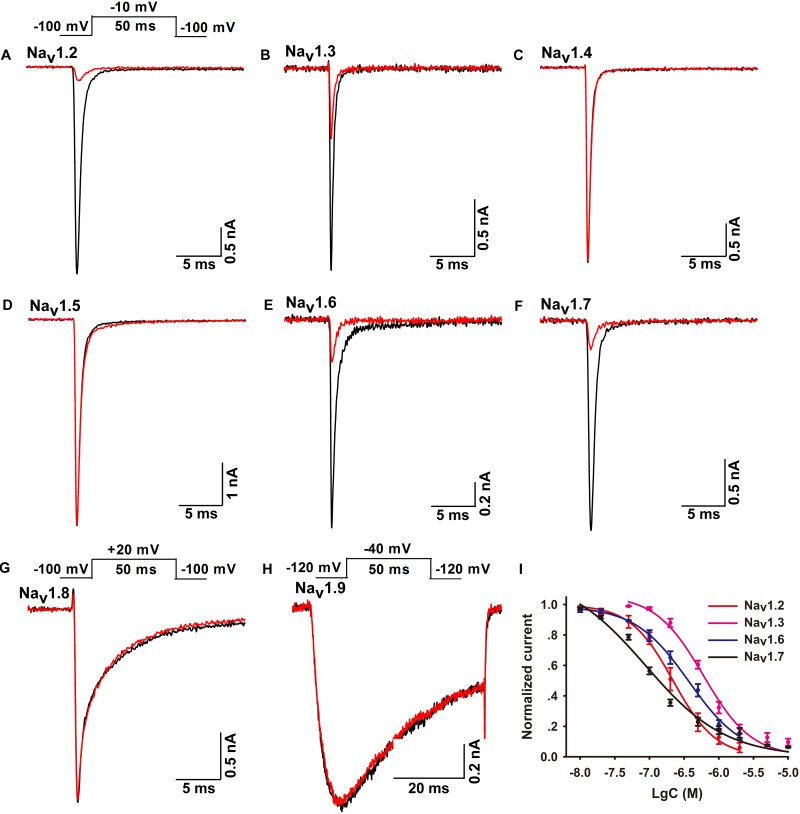
Effects of μ-TRTX-Ca2a on Na_v_1.2–Na_v_1.9 channels. **(A–H)** Representative Na_v_1.2–Na_v_1.9 current traces before (black) and after (red) addition of Ca2a. Ca2a at 1 μM inhibited Na_v_1.2–Na_v_1.3, Na_v_1.6, and Na_v_1.7. 10 μM Ca2a showed no obvious effect on Na_v_1.4–Na_v_1.5 or Na_v_1.8–Na_v_1.9 current. Inset above panel **(A)** shows the pulse protocol for recording Na_v_1.2–Na_v_1.7 channel currents. Inset above panel **(G)** shows the pulse protocol for recording Na_v_1.8 channel current. Inset above panel **(H)** shows the pulse protocol for recording Na_v_1.9 channel current. **(I)** Concentration-response curves of Ca2a at Na_v_1.2–Na_v_1.3, Na_v_1.6, and Na_v_1.7 assessed by whole-cell patch-clamp experiments. Data are mean ± SEM, with *n* = 4–7 cells per data point.

The biological function of Ca2a was investigated on HEK293T cells transiently transfected with VGSCs. A total of 1 μM Ca2a showed 87.3 ± 4.4% inhibition on Na_v_1.7 currents, and decreased the Na_v_1.2, Na_v_1.3, and Na_v_1.6 current amplitude by 82.6 ± 2.7%, 67.7 ± 3.8%, and 78.7 ± 2.9%, respectively. However, no inhibitory effects were observed against Na_v_1.4, Na_v_1.5, Na_v_1.8, or Na_v_1.9 currents even at high concentrations of up to 10 μM Ca2a (**Figures 2A–H**). Currents of Na_v_1.1 were not detected in heterogeneously expressed HEK293T cells, and the effect of Ca2a on Na_v_1.1 could not be examined in the present study. Thus, Ca2a had highest potency for Na_v_1.7 (IC_50_ of 98.1 ± 3.3 nM), followed by Na_v_1.2 (IC_50_ of 216.3 ± 9.1 nM), Na_v_1.6 (IC_50_ of 313.6 ± 6.3 nM), and Na_v_1.3 (IC_50_ of 491.3 ± 3.9 nM) (**Figure 2I**).

### Effect of Ca2a on Na_v_1.7 Activation and Inactivation Properties

**FIGURE 3:**
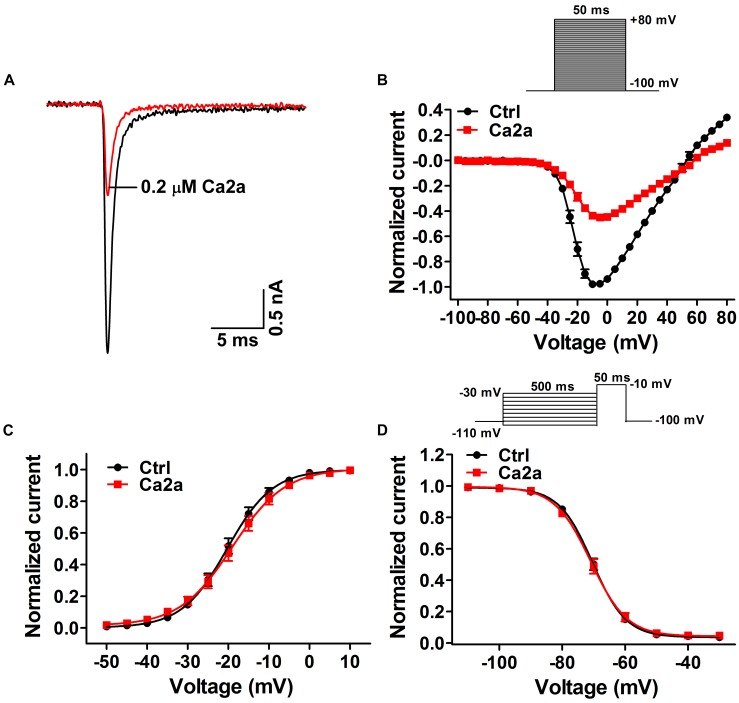
Effects of μ-TRTX-Ca2a on the voltage dependence of Na_v_1.7 activation and inactivation gating. **(A)** Representative current traces of Na_v_1.7 channel inhibited by 0.2 μM Ca2a. **(B)** I–V curves before (black) and after (red) treatment of Ca2a (*n* = 10). Inset shows the pulse protocol for measuring current–voltage (I–V) relationships. **(C)** G–V curves before (black) and after (red) treatment of Ca2a (*n* = 10). **(D)** Voltage-dependence of steady-state fast inactivation curves before (black) and (red) after treatment of Ca2a (*n* = 10). Inset shows the pulse protocol for measuring steady-state fast inactivation.

Many spider peptide toxins are regarded as gating modifiers because these toxins bind to the voltage-sensing domains of Na_v_ channels and alter voltage dependence of activation and/or inactivation (Catterall et al., 2007). Ca2a inhibited 64.5 ± 2.1% of the Na_v_1.7 current at the concentration of 0.2 μM (**Figure 3A**), so we chose this subsaturation concentration to analyze the effects of Ca2a on the activation and inactivation of Na_v_1.7. Ca2a decreased the currents at all tested voltages, but it did not change the threshold of initial activation voltage, the active voltage of peak current, or the reversal potential of the Na_v_1.7 current (**Figure 3B**). In addition, the half-activation voltage and half-inactivation voltage of Na_v_1.7 after treatment with 0.2 μM Ca2a were −18.9 ± 1.3 and −70.7 ± 1.1 mV, respectively. In the control group, the half-activation voltage and the half-inactivation voltage of Na_v_1.7 were −20.1 ± 1.1 and −70.2 ± 0.8 mV, respectively. These results indicated that Ca2a inhibited the peak currents without affecting the voltage-dependent activation and fast inactivation (**Figures 3C,D**).

### Kinetics of Ca2a Inhibition and Dissociation in Na_v_1.7

**FIGURE 4:**
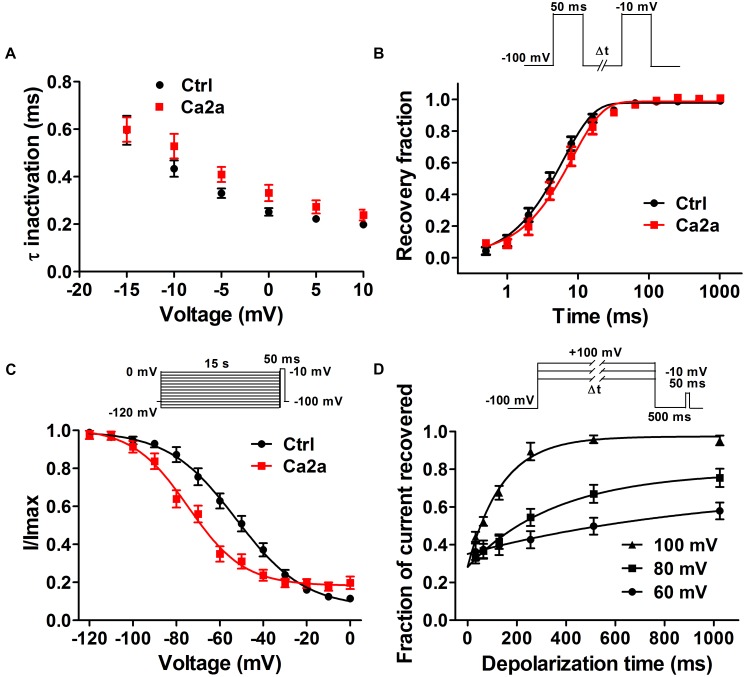
Na_v_1.7 kinetic parameters affected by the addition of μ-TRTX-Ca2a. **(A)** Voltage-dependence of fast inactivation time constants of Na_v_1.7 before (black) and after (red) addition of 0.2 μM Ca2a. **(B)** Kinetics of current recovery from fast inactivation of Na_v_1.7 before (black) and after (red) addition of 0.2 μM Ca2a at –100 mV. Inset shows the pulse protocol for measuring recovery from fast-inactivation. **(C)** Voltage-dependence of steady-state slow inactivation of Na_v_1.7 before (black) and after (red) addition of 0.2 μM Ca2a. Inset shows the pulse protocol for measuring steady-state slow inactivation. **(D)** Time course of dissociation of 1 μM Ca2a from Na_v_1.7 at 100, 80, and 60 mV. Data are mean ± SEM, with *n* = 6–11 cells per data point. Inset shows the pulse protocol for measuring the rate of toxin dissociation.

Spider toxins usually modulate the gating behaviors of VGSCs and can influence voltage sensor movement (Yamaji et al., 2009; Bosmans and Swartz, 2010). To investigate the effects of Ca2a on the kinetics of fast inactivation and repriming, we measured the time constants of fast inactivation and recovery from fast inactivation. Fast inactivation time constants were calculated by fitting current decay traces with a single exponential function. Ca2a only inhibited the peak current, but it did not alter the inactivation time constants between −15 and 10 mV (*P* > 0.05; two-way ANOVA*;* **Figure 4A**). However, Ca2a significantly but modestly slowed recovery from fast inactivation at Na_v_1.7 (control, τ = 6.42 ± 0.85 ms; Ca2a, τ = 8.93 ± 1.62 ms; *P* < 0.05*;* paired *t*-test*;* **Figure 4B**). Moreover, Ca2a significantly shifted steady-state slow inactivation to more hyperpolarized membrane potentials (control, −52.62 ± 3.79 mV; Ca2a, −72.00 ± 2.76 mV; *P* < 0.01; paired *t*-test; **Figure 4C**). Slow inactivation is a process that occurs under a high-frequency stimulation or a prolonged depolarizing pulse. This process is most likely to involve a rearrangement of the channel pore, which results in a different conformational state and directly regulates cellular excitability (Chatterjee et al., 2018). The dissociation time constants were calculated to be 169.8 ± 25.6 ms at 100 mV, 347.9 ± 29.2 ms at 80 mV, and 547.2 ± 24.3 ms at 60 mV in the presence of 1 μM Ca2a (**Figure 4D**). This indicated that Ca2a dissociated quickly from Na_v_1.7 in a voltage-dependent manner. In addition, Ca2a did not show obvious use/frequency dependence of inhibition for Na_v_1.7, and the IC_50_ values of 1, 5, and 10 Hz were 229.3 ± 34.8, 243.8 ± 30.7, and 184.9 ± 23.8 nM, respectively (**Supplementary Figure S1**).

### Ca2a Binds to the DIIS3–S4 Linker of Na_v_1.7

**FIGURE 5:**
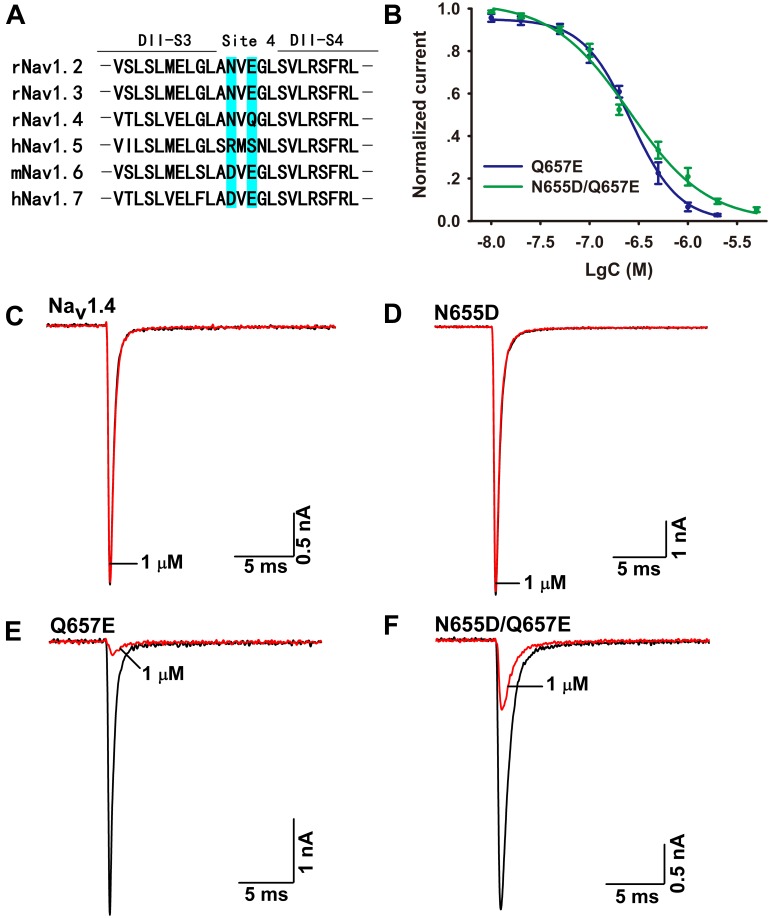
Effect of Ca2a on WT and mutant Na_v_1.4 expressed in HEK293T cells. **(A)** Amino acids of potential binding sites in the sequence were shaded in *cyan*. Sequence alignment of DIIS3-S4 of Na_v_1.2–Na_v_1.7. **(B)** Dose-response curves of Q657E and N655D/Q657E. **(C–F)** Representative current traces for WT and mutant channels (N655D, Q657E, and N655D/Q657E) inhibited by 1 μM Ca2a. Data are mean ± SEM, with *n* = 4–5 cells per data point.

The mechanism of Ca2a acting on Na_v_1.7 is similar to that of HWTX-IV and HNTX-III, and it might be a site 4 toxin acting on the DIIS3–S4 linker of the sodium channel (Xiao et al., 2008; Liu et al., 2013). As Ca2a showed no activity on Na_v_1.4, we constructed chimeric channels of Na_v_1.4 and Na_v_1.7 to validate this hypothesis. As shown in **Figure 5A**, only two amino acids are different in DIIS3–S4 linkers of Na_v_1.4 and Na_v_1.7. To investigate the role of these two acidic residues, we mutated their compartments in Na_v_1.4 (N655D, Q657E, and N655D/Q657E). The results showed that 1 μM Ca2a had no inhibitory effect on WT Na_v_1.4 and Na_v_1.4/N655D, but it significantly inhibited the peak current of Na_v_1.4/Q657E and Na_v_1.4/N655D/Q657E (**Figures 5C–F**). The IC_50_ values of Na_v_1.4/Q657E and Nav1.4/N655D/Q657E were 268.9 ± 14.9 and 237.9 ± 26.1 nM, respectively (**Figure 5B**). These results indicate that the residue Q657 plays an important role in resistance to Ca2a rather than N655 located in DIIS3–S4 linker of Na_v_1.4.

**FIGURE 6:**
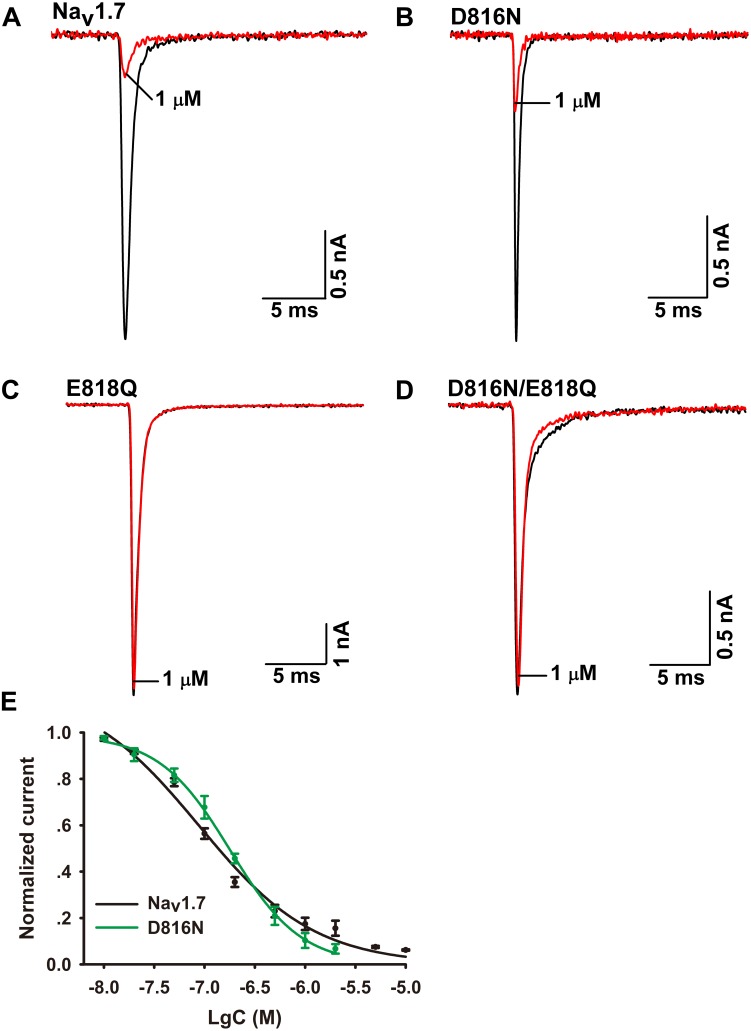
Effect of Ca2a on WT and mutant Na_v_1.7 expressed in HEK293T cells. **(A)** Representative current traces for WT inhibited by 1 μM Ca2a. **(B–D)** Representative current traces for mutant channels (D816N, E818Q, and D816N/E818Q) inhibited by 1 μM Ca2a. **(E)** Dose-response curves of Na_v_1.7 and D816N. Data are mean ± SEM, with *n* = 5 cells per data point.

To validate the role of Q657 in Ca2a inhibition, we constructed reverse mutations in Na_v_1.7 (D816N, E818Q, and D816N/E818Q). A total of 1 μM Ca2a significantly inhibited Na_v_1.7/D816N current amplitude with IC_50_ value (172.0 ± 11.4 nM) twofold higher than the wild type. This validates the hypothesis that D816 plays a negligible role in Ca2a interacting with Na_v_1.7 (**Figures 6B,E**). The time course of 1 μM Ca2a inhibiting the Nav1.7/D816N current was characterized by a slow onset of action (τ_*on*_ = 18.4 ± 1.3 s) similar to that of WT Na_v_1.7 (τ_*on*_ = 18.0 ± 2.5 s) while the current did not recover during the extended washout in contrast to that of WT Na_v_1.7 (τ_*off*_ = 295.6 ± 27.5 s) (**Supplementary Figures S2A,B**). In contrast to complete inhibition on WT Na_v_1.7 (**Figure 6A**), 1 μM Ca2a showed no activity on Na_v_1.7/E818Q or Na_v_1.7/D816N/E818Q, implying that E818 plays an important role in Ca2a inhibition (**Figures 6C,D**). These results demonstrate that the DIIS3–S4 linker is critical for Ca2a binding to the sodium channel.

### Effects of Ca2a on Pain

To assess the analgesic potential of Ca2a *in vivo*, we examined the effects of Ca2a in animal models of pain including formalin-induced paw licking, hot plate test and acetic acid-induced writhing.

**FIGURE 7:**
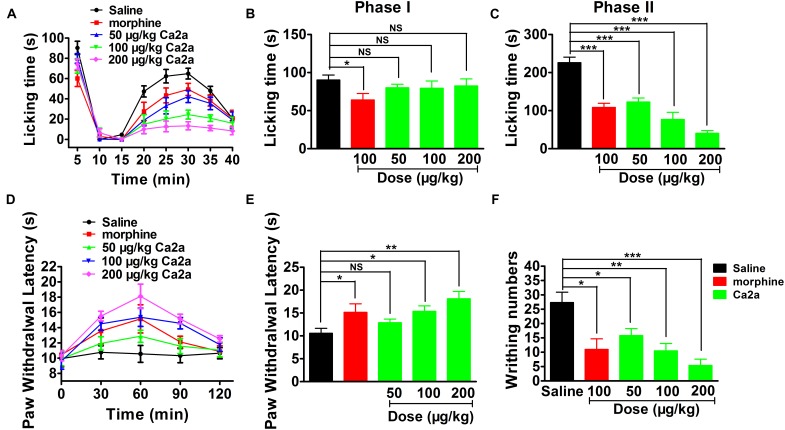
Analgesic effect of Ca2a. **(A)** Time course of the antinociceptive effect of Ca2a in the formalin test. Evaluation of the antinociceptive effect of Ca2a on phase I **(B)** or phase II **(C)**. **(D)** Time course of the antinociceptive effect of Ca2a in hot plate test. **(E)** Analgesic effect was assessed after 30 min of Ca2a injection. **(F)** The antinociceptive effect of Ca2a in the abdominal constriction test. The data are shown as mean ± SEM, with *n* = 6–8; ^∗^*P* < 0.05, ^∗∗^*P* < 0.01, ^∗∗∗^*P* < 0.001 vs. vehicle.

The paw licking time of the control was 90.3 ± 6.6 s on Phase I (0–15 min) and 197.7 ± 20.7 s on Phase II (15–40 min) (**Figure 7A**). The paw licking time in Phase I was 80.1 ± 4.6 s, 79.4 ± 9.5 s and 75.6 ± 7.7 s for 50, 100, and 200 μg/kg Ca2a, respectively, while the paw licking time of 100 μg/kg morphine was 64.1 ± 8.5 s on Phase I (**Figure 7A**). Ca2a (50, 100, and 200 μg/kg) produced no analgesic effect on Phase I compared to the control while morphine (100 μg/kg) showed modest analgesia (**Figure 7B**). However, Ca2a produced a significant analgesic effect in a dose-dependent manner on Phase II. The paw licking time was significantly reduced to 100.9 ± 16.2 s, 67.2 ± 20.2 s, and 40.5 ± 7.1 s for 50, 100, and 200 μg/kg Ca2a, respectively. The paw licking time of 100 μg/kg morphine was 126.1 ± 19.8 s in Phase II (**Figure 7C**). In the hot plate test, Ca2a also showed a strong analgesic effect (**Figure 7D**). The latency time of the control was 10.6 ± 1.1 s, while the latency time of Ca2a at each dose (50, 100, and 200 μg/kg) was increased to 12.9 ± 0.8 s, 15.4 ± 1.2 s, and 18.1 ± 1.6 s, respectively. As a positive control, the latency time of morphine at a concentration of 2 mg/kg increased to 15.2 ± 1.9 s (**Figure 7E**). In the acetic acid-induced writhing test, Ca2a dose-dependently reduced the writhing numbers. Intraperitoneal injection of 50, 100, and 200 μg/kg Ca2a reduced the duration of writhing from 27.3 ± 3.3 of the control to 15.8 ± 2.4, 10.5 ± 2.8, and 5.4 ± 2.2, respectively, while morphine at 100 μg/kg caused a reduction to 11 ± 3.7 (**Figure 7F**).

## Discussion

In this study, we described the identification and characterization of a novel peptide μ-TRTX-Ca2a, which is a 35-residue peptide toxin isolated from the venom of tarantula spider *C. albostriatus* with six cysteines and belongs to the ICK motif. It has been thought that ICK toxins typically owned tremendous chemical, thermal, and biological stability and provided a variety of delivery options for therapeutic administration (Colgrave and Craik, 2004; Saez et al., 2010). Ca2a inhibited Na_v_1.2, Na_v_1.3, Na_v_1.6, and Na_v_1.7 channels but had negligible effect on Na_v_1.4, Na_v_1.5, Na_v_1.8, and Na_v_1.9 channels, suggesting that Ca2a is a selective antagonist of neuronal TTX-S VGSCs. Meanwhile, Ca2a ought to have an effect on neurons where Na_v_1.7 accounts for a majority of TTX-S Na^+^ current, and peptide toxins inhibiting Na_v_1.7 usually have inhibitory activity on DRG neurons (Deuis et al., 2017; Kornecook et al., 2017; Moyer et al., 2018).

Ca2a belongs to NaSpTx family 1 and shares identity to some known spider toxins. β-TRTX-Hlv1a from *Haplopelma lividum* exhibits 94.3% identity to Ca2a (Meir et al., 2011). Although β-TRTX-Hlv1a was thought to be a Na_v_ channel inhibitor, it only inhibited Na_v_1.3 with IC_50_ of 1 μM. Moreover, β-TRTX-Ps1a (Pautx-3) and ω-TRTX-Gr2a (GpTx-1) with an identity of 68.8 and 62.9% to Ca2a, respectively, were potent VGSC blockers (Bosmans et al., 2006; Cherki et al., 2014). β-TRTX-Ps1a was an inhibitor of Na_v_1.2, but it had no effect on Na_v_1.7. ω-TRTX-Gr2a exhibited activity against all sodium channel subtypes (Na_v_1.1–Na_v_1.8) without any selectivity. Similar to μ-TRTX-Hhn1b (HWTX-IV), Ca2a showed preferred affinity to Na_v_1.7 without inhibitory activity against Na_v_1.5 or Na_v_1.4 while HWTX-IV inhibited skeletal isoform Na_v_1.4 with an IC_50_ value of 400 nM (Xiao et al., 2008; Goncalves et al., 2018). Therefore, Ca2a shows stronger activity or higher selectivity to Na_v_1.7 than other similar toxins.

Consistent with the molecular mechanism of μ-TRTX-Hd1a, μ-TRTX-Hhn1b (HNTX-IV), and μ-TRTX-Hs2a (HWTX-IV) interacting with Na_v_1.7, Ca2a caused no change of I–V curve, G–V curve, and steady-state fast inactivation. The critical residue of Ca2a binding to Na_v_1.7 channel is E818 in the S3–S4 linker of DII. E818 also plays an important role in HWTX-IV’s binding, but another residue D816 critical for HWTX-IV binding does not (Xiao et al., 2008; Cai et al., 2015; Klint et al., 2015). However, E818 is a critical residue for Ca2a binding to Na_v_1.7, and Na_v_1.7/E818Q greatly reduces the binding affinity of Ca2a. The IC_50_ value of Na_v_1.7/D816N is similar to that of wild-type Na_v_1.7, implying that D816 does not play an important role in binding affinity. Asn (Na_v_1.7/D816N) residue may strengthen the binding energy and make the binding a more compact. The irreversible washout of Ca2a inhibition of Na_v_1.7/D816N may be attributed to the higher binding energy and more compact structure. Ca2a did not affect channel inactivation progress, which was validated by a similar inactivation process observed in Na_v_1.7/D816N channel (**Supplementary Figure S3**). Nevertheless, Ca2a significantly but modestly slowed recovery from fast inactivation. In addition, the large hyperpolarized shift in slow inactivation suggests that Ca2a binds tightly to the slow inactivated state of the channel. These results suggest Ca2a may also interact with DIV because toxins affect channel inactivation by interacting with DIV (Xiao et al., 2010; Tao et al., 2016), and activation is affected by toxins binding to any of DI–III (Klint et al., 2015). This is similar with ProTx-II in which DII and DIV voltage sensors are involved in interaction by the dual modulatory effect (Bosmans et al., 2008) and a recently reported peptide Pn3a interacting with DII and DIV voltage sensors (Deuis et al., 2017). The mechanism of Ca2a acting on Na_v_1.7 is similar to that of HWTX-IV, but it also exhibits a little difference.

In formalin-induced paw licking, hot plate test and abdominal writhing, Ca2a showed dose-dependently equipotent or stronger analgesia than morphine (100 μg/kg Ca2a equals to the concentration of 25.6 nmol/kg Ca2a, and 100 μg/kg morphine refers to 350.9 nmol/kg morphine). As a selective antagonist of neuronal TTX-S VGSCs, Ca2a preferentially inhibited Na_v_1.7 with more than 100-fold selectivity against off-targets skeletal isoform Na_v_1.4 and cardiac isoform Na_v_1.5. Of the nine mammalian sodium channels, Na_v_1.3, Na_v_1.7, Na_v_1.8, and Na_v_1.9 channel subtypes are widely regarded as “pain channels” associated with nociception and chronic pain disorders that play essential roles in pain pathway (Dib-Hajj et al., 2010; Liu and Wood, 2011). In addition, Na_v_1.2, distributed in the central nervous system, is unlikely to contribute to analgesia because it is associated with epilepsy (Sugawara et al., 2001). Na_v_1.6, mainly distributed in the central nervous system and mature nodes of Ranvier in the peripheral nervous system (Habib et al., 2015), has been previously shown to be related with infantile epileptic encephalopathy (Veeramah et al., 2012; Estacion et al., 2014; Blanchard et al., 2015). However, recent studies have reported that the gain-of-function mutation of Na_v_1.6 increased trigeminal ganglia (TRG) neuron excitability in trigeminal neuralgia (Tanaka et al., 2016) while Na_v_1.6 knockdown ameliorated mechanical pain behavior in models of local inflammation and neuropathic pain (Xie et al., 2013, 2015). The exact role of Na_v_1.6 in the pain pathway currently remains unclear currently. Inhibition of Na_v_1.6 was also previously regarded to cause movement disorders and hind limb paralysis (Meisler et al., 2001). The dose of Ca2a used in this study does not cause significant adverse effects, indicating that the peptide dose not target Na_v_1.6 *in* *vivo.* Meanwhile, the expression of Na_v_1.7 is higher than other TTX-S VGSCs in DRG neurons (Fukuoka et al., 2008; Ho and O’Leary, 2011). Na_v_1.3 is not expressed in adult rat DRG except for upregulated expression after nerve injury (Abe et al., 2002). Meanwhile, the effective analgesic doses of Ca2a used in the studies are very low. These results suggest that the analgesic effect of Ca2a is due to the inhibition of Na_v_1.7 and not affected by blocking other Na_v_ channels. The effect of Ca2a on motor functions remains to be elucidated, and further study is required to improve the selectivity and potency of Ca2a, making Ca2a a clinical potential peptide for the treatment of pain.

In summary, a new spider peptide toxin named μ-TRTX-Ca2a was identified. Ca2a binds to the DIIS3–S4 linker to inhibit channel current and may interact with DIVS3–S4 to affect channel inactivation. Moreover, *in* *vivo* analgesic efficacy suggests Ca2a may be a lead molecule for the development of analgesics targeting Na_v_1.7 channel.

## Author Contributions

YZ, MR, and ZL conceived and designed the experiments. YZ, DP, BH, QY, QZ, and MC performed the experiments. YZ, DP, BH, and MR analyzed the data. YZ and MR wrote the manuscript. All authors have read and approved the manuscript.

## Conflict of Interest Statement

The authors declare that the research was conducted in the absence of any commercial or financial relationships that could be construed as a potential conflict of interest.

## References

[B1] Abe M. Kurihara T. Han W. Shinomiya K. Tanabe T. (2002). Changes in expression of voltage-dependent ion channel subunits in dorsal root ganglia of rats with radicular injury and pain. *Spine* 27 1517–1524; discussion 1525. 12131710 10.1097/00007632-200207150-00007

[B2] Blanchard M. G. Willemsen M. H. Walker J. B. Dib-Hajj S. D. Waxman S. G. Jongmans M. C. (2015). De novo gain-of-function and loss-of-function mutations of SCN8A in patients with intellectual disabilities and epilepsy. *J. Med. Genet.* 52 330–337. 10.1136/jmedgenet-2014-102813 25725044 PMC4413743

[B3] Bosmans F. Martin-Eauclaire M. F. Swartz K. J. (2008). Deconstructing voltage sensor function and pharmacology in sodium channels. *Nature* 456 202–208. 10.1038/nature07473 19005548 PMC2587061

[B4] Bosmans F. Rash L. Zhu S. Diochot S. Lazdunski M. Escoubas P. (2006). Four novel tarantula toxins as selective modulators of voltage-gated sodium channel subtypes. *Mol. Pharmacol.* 69 419–429. 10.1124/mol.105.015941 16267209

[B5] Bosmans F. Swartz K. J. (2010). Targeting voltage sensors in sodium channels with spider toxins. *Trends Pharmacol. Sci.* 31 175–182. 10.1016/j.tips.2009.12.007 20097434 PMC2847040

[B6] Cai T. Luo J. Meng E. Ding J. Liang S. Wang S. (2015). Mapping the interaction site for the tarantula toxin hainantoxin-IV (beta-TRTX-Hn2a) in the voltage sensor module of domain II of voltage-gated sodium channels. *Peptides* 68 148–156. 10.1016/j.peptides.2014.09.005 25218973

[B7] Catterall W. A. (2000). From ionic currents to molecular mechanisms: the structure and function of voltage-gated sodium channels. *Neuron* 26 13–25. 10.1016/S0896-6273(00)81133-2 10798388

[B8] Catterall W. A. (2012). Voltage-gated sodium channels at 60: structure, function and pathophysiology. *J. Physiol.* 590 2577–2589. 10.1113/jphysiol.2011.224204 22473783 PMC3424717

[B9] Catterall W. A. (2014). Structure and function of voltage-gated sodium channels at atomic resolution. *Exp. Physiol.* 99 35–51. 10.1113/expphysiol.2013.071969 24097157 PMC3885250

[B10] Catterall W. A. Cestele S. Yarov-Yarovoy V. Yu F. H. Konoki K. Scheuer T. (2007). Voltage-gated ion channels and gating modifier toxins. *Toxicon* 49 124–141. 10.1016/j.toxicon.2006.09.022 17239913

[B11] Chatterjee S. Vyas R. Chalamalasetti S. V. Sahu I. D. Clatot J. Wan X. (2018). The voltage-gated sodium channel pore exhibits conformational flexibility during slow inactivation. *J. Gen. Physiol.* 150 1333–1347. 10.1085/jgp.201812118 30082431 PMC6122925

[B12] Cherki R. S. Kolb E. Langut Y. Tsveyer L. Bajayo N. Meir A. (2014). Two tarantula venom peptides as potent and differential Na(V) channels blockers. *Toxicon* 77 58–67. 10.1016/j.toxicon.2013.10.029 24211312

[B13] Colgrave M. L. Craik D. J. (2004). Thermal, chemical, and enzymatic stability of the cyclotide kalata B1: the importance of the cyclic cystine knot. *Biochemistry* 43 5965–5975. 10.1021/bi049711q 15147180

[B14] Deuis J. R. Dekan Z. Wingerd J. S. Smith J. J. Munasinghe N. R. Bhola R. F. (2017). Pharmacological characterisation of the highly NaV1.7 selective spider venom peptide Pn3a. *Sci. Rep.* 7:40883. 10.1038/srep40883 28106092 PMC5247677

[B15] Dib-Hajj S. D. Black J. A. Waxman S. G. (2009). Voltage-gated sodium channels: therapeutic targets for pain. *Pain Med.* 10 1260–1269. 10.1111/j.1526-4637.2009.00719.x 19818036

[B16] Dib-Hajj S. D. Cummins T. R. Black J. A. Waxman S. G. (2010). Sodium channels in normal and pathological pain. *Annu. Rev. Neurosci.* 33 325–347. 10.1146/annurev-neuro-060909-153234 20367448

[B17] Escoubas P. King G. F. (2009). Venomics as a drug discovery platform. *Expert Rev. Proteomics* 6 221–224. 10.1586/epr.09.45 19489692

[B18] Estacion M. O’Brien J. E. Conravey A. Hammer M. F. Waxman S. G. Dib-Hajj S. D. (2014). A novel de novo mutation of SCN8A (Nav1.6) with enhanced channel activation in a child with epileptic encephalopathy. *Neurobiol. Dis.* 69 117–123. 10.1016/j.nbd.2014.05.017 24874546 PMC4124819

[B19] Faber C. G. Hoeijmakers J. G. Ahn H. S. Cheng X. Han C. Choi J. S. (2012). Gain of function Nav1. 7 mutations in idiopathic small fiber neuropathy. *Ann. Neurol.* 71 26–39. 10.1002/ana.22485 21698661

[B20] Fertleman C. R. Baker M. D. Parker K. A. Moffatt S. Elmslie F. V. Abrahamsen B. (2006). SCN9A mutations in paroxysmal extreme pain disorder: allelic variants underlie distinct channel defects and phenotypes. *Neuron* 52 767–774. 10.1016/j.neuron.2006.10.006 17145499

[B21] Fukuoka T. Kobayashi K. Yamanaka H. Obata K. Dai Y. Noguchi K. (2008). Comparative study of the distribution of the alpha-subunits of voltage-gated sodium channels in normal and axotomized rat dorsal root ganglion neurons. *J. Comp. Neurol.* 510 188–206. 10.1002/cne.21786 18615542

[B22] Gingras J. Smith S. Matson D. J. Johnson D. Nye K. Couture L. (2014). Global Nav1. 7 knockout mice recapitulate the phenotype of human congenital indifference to pain. *PLoS One* 9:e105895. 10.1371/journal.pone.0105895 25188265 PMC4154897

[B23] Goncalves T. C. Boukaiba R. Molgo J. Amar M. Partiseti M. Servent D. (2018). Direct evidence for high affinity blockade of NaV1.6 channel subtype by huwentoxin-IV spider peptide, using multiscale functional approaches. *Neuropharmacology* 133 404–414. 10.1016/j.neuropharm.2018.02.016 29474819

[B24] Habib A. M. Wood J. N. Cox J. J. (2015). Sodium channels and pain. *Handb. Exp. Pharmacol.* 227 39–56. 10.1007/978-3-662-46450-2-3 25846613

[B25] Ho C. O’Leary M. E. (2011). Single-cell analysis of sodium channel expression in dorsal root ganglion neurons. *Mol. Cell. Neurosci.* 46 159–166. 10.1016/j.mcn.2010.08.017 20816971 PMC3005531

[B26] King G. F. (2011). Venoms as a platform for human drugs: translating toxins into therapeutics. *Expert Opin. Biol. Ther.* 11 1469–1484. 10.1517/14712598.2011.621940 21939428

[B27] Klint J. K. Senff S. Rupasinghe D. B. Er S. Y. Herzig V. Nicholson G. M. (2012). Spider-venom peptides that target voltage-gated sodium channels: pharmacological tools and potential therapeutic leads. *Toxicon* 60 478–491. 10.1016/j.toxicon.2012.04.337 22543187

[B28] Klint J. K. Smith J. J. Vetter I. Rupasinghe D. B. Er S. Y. Senff S. (2015). Seven novel modulators of the analgesic target NaV 1.7 uncovered using a high-throughput venom-based discovery approach. *Br. J. Pharmacol.* 172 2445–2458. 10.1111/bph.13081 25754331 PMC4409898

[B29] Kornecook T. J. Yin R. Altmann S. Be X. Berry V. Ilch C. P. (2017). Pharmacologic characterization of AMG8379, a potent and selective small molecule sulfonamide antagonist of the voltage-gated sodium channel NaV1.7. *J. Pharmacol. Exp. Ther.* 362 146–160. 10.1124/jpet.116.239590 28473457

[B30] Liu M. Wood J. N. (2011). The roles of sodium channels in nociception: implications for mechanisms of neuropathic pain. *Pain Med.* 12(Suppl. 3), S93–S99. 10.1111/j.1526-4637.2011.01158.x 21752183

[B31] Liu Y. Tang J. Zhang Y. Xun X. Tang D. Peng D. (2014). Synthesis and analgesic effects of mu-TRTX-Hhn1b on models of inflammatory and neuropathic pain. *Toxins* 6 2363–2378. 10.3390/toxins6082363 25123556 PMC4147587

[B32] Liu Z. Cai T. Zhu Q. Deng M. Li J. Zhou X. (2013). Structure and function of hainantoxin-III, a selective antagonist of neuronal tetrodotoxin-sensitive voltage-gated sodium channels isolated from the Chinese bird spider *Ornithoctonus hainana*. *J. Biol. Chem.* 288 20392–20403. 10.1074/jbc.M112.426627 23703613 PMC3711305

[B33] Mansouri M. Elalaoui S. C. Bencheikh B. O. A. El Alloussi M. Dion P. A. Sefiani A. (2014). A novel nonsense mutation in SCN9A in a moroccan child with congenital insensitivity to pain. *Pediatr. Neurol.* 51 741–744. 10.1016/j.pediatrneurol.2014.06.009 25439579

[B34] Meir A. Cherki R. S. Kolb E. Langut Y. Bajayo N. (2011). Novel peptides isolated from spider venom, and uses thereof. U. S. Patent No US 2011/0065647A1. Washington, DC: U. S. Patent and Trademark Office.

[B35] Meisler M. H. Kearney J. Escayg A. MacDonald B. T. Sprunger L. K. (2001). Sodium channels and neurological disease: insights from Scn8a mutations in the mouse. *Neuroscientist* 7 136–145. 10.1177/107385840100700208 11496924

[B36] Meng D. Wang L. Du J. Chen J. Chen C. Xu W. (2017). The analgesic activities of *Stauntonia brachyanthera* and YM 11 through regulating inflammatory mediators and directly controlling the sodium channel prompt. *Sci. Rep.* 7:7574. 10.1038/s41598-017-07095-x 28790377 PMC5548894

[B37] Moyer B. D. Murray J. K. Ligutti J. Andrews K. Favreau P. Jordan J. B. (2018). Pharmacological characterization of potent and selective NaV1.7 inhibitors engineered from *Chilobrachys jingzhao* tarantula venom peptide JzTx-V. *PLoS One* 13:e0196791. 10.1371/journal.pone.0196791 29723257 PMC5933747

[B38] Owoyele V. B. Adediji J. O. Soladoye A. O. (2005). Anti-inflammatory activity of aqueous leaf extract of *Chromolaena odorata*. *Inflammopharmacology* 13 479–484. 10.1163/156856005774649386 16280100

[B39] Saez N. J. Senff S. Jensen J. E. Er S. Y. Herzig V. Rash L. D. (2010). Spider-venom peptides as therapeutics. *Toxins* 2 2851–2871. 10.3390/toxins2122851 22069579 PMC3153181

[B40] Shorer Z. Wajsbrot E. Liran T.-H. Levy J. Parvari R. (2014). A novel mutation in SCN9A in a child with congenital insensitivity to pain. *Pediatr. Neurol.* 50 73–76. 10.1016/j.pediatrneurol.2013.09.007 24188911

[B41] Sugawara T. Tsurubuchi Y. Agarwala K. L. Ito M. Fukuma G. Mazaki-Miyazaki E. (2001). A missense mutation of the Na + channel alpha II subunit gene Na(v)1.2 in a patient with febrile and afebrile seizures causes channel dysfunction. *Proc. Natl. Acad. Sci. U.S.A.* 98 6384–6389. 10.1073/pnas.111065098 11371648 PMC33477

[B42] Tanaka B. S. Zhao P. Dib-Hajj F. B. Morisset V. Tate S. Waxman S. G. (2016). A gain-of-function mutation in Nav1.6 in a case of trigeminal neuralgia. *Mol. Med.* 22 338–348. 10.2119/molmed.2016.00131 27496104 PMC5023517

[B43] Tao H. Chen X. Lu M. Wu Y. Deng M. Zeng X. (2016). Molecular determinant for the tarantula toxin Jingzhaotoxin-I slowing the fast inactivation of voltage-gated sodium channels. *Toxicon* 111 13–21. 10.1016/j.toxicon.2015.12.009 26721415

[B44] Veeramah K. R. O’Brien J. E. Meisler M. H. Cheng X. Dib-Hajj S. D. Waxman S. G. (2012). De novo pathogenic SCN8A mutation identified by whole-genome sequencing of a family quartet affected by infantile epileptic encephalopathy and SUDEP. *Am. J. Hum. Genet.* 90 502–510. 10.1016/j.ajhg.2012.01.006 22365152 PMC3309181

[B45] Wang W. Gu J. Li Y.-Q. Tao Y.-X. (2011). Are voltage-gated sodium channels on the dorsal root ganglion involved in the development of neuropathic pain? *Mol. Pain* 7:16. 10.1186/1744-8069-7-16 21345196 PMC3052185

[B46] Wu B. Zhang Y. Tang H. Yang M. Long H. Shi G. (2017). A novel SCN9A mutation (F826Y) in primary erythromelalgia alters the excitability of Nav1. 7. *Curr. Mol. Med.* 17 450–457. 10.2174/1566524017666171009105029 28990532

[B47] Xiao Y. Bingham J. P. Zhu W. Moczydlowski E. Liang S. Cummins T. R. (2008). Tarantula huwentoxin-IV inhibits neuronal sodium channels by binding to receptor site 4 and trapping the domain ii voltage sensor in the closed configuration. *J. Biol. Chem.* 283 27300–27313. 10.1074/jbc.M708447200 18628201 PMC2556013

[B48] Xiao Y. Blumenthal K. Jackson J. O. II Liang S. Cummins T. R. (2010). The tarantula toxins ProTx-II and huwentoxin-IV differentially interact with human Nav1.7 voltage sensors to inhibit channel activation and inactivation. *Mol. Pharmacol.* 78 1124–1134. 10.1124/mol.110.066332 20855463 PMC2993464

[B49] Xie W. Strong J. A. Ye L. Mao J. X. Zhang J. M. (2013). Knockdown of sodium channel NaV1.6 blocks mechanical pain and abnormal bursting activity of afferent neurons in inflamed sensory ganglia. *Pain* 154 1170–1180. 10.1016/j.pain.2013.02.027 23622763 PMC3699898

[B50] Xie W. Strong J. A. Zhang J. M. (2015). Local knockdown of the NaV1.6 sodium channel reduces pain behaviors, sensory neuron excitability, and sympathetic sprouting in rat models of neuropathic pain. *Neuroscience* 291 317–330. 10.1016/j.neuroscience.2015.02.010 25686526 PMC4369447

[B51] Yamaji N. Little M. J. Nishio H. Billen B. Villegas E. Nishiuchi Y. (2009). Synthesis, solution structure, and phylum selectivity of a spider delta-toxin that slows inactivation of specific voltage-gated sodium channel subtypes. *J. Biol. Chem.* 284 24568–24582. 10.1074/jbc.M109.030841 19592486 PMC2782047

[B52] Zhou X. Xiao Z. Xu Y. Zhang Y. Tang D. Wu X. (2017). Electrophysiological and pharmacological analyses of Nav1.9 voltage-gated sodium channel by establishing a heterologous expression system. *Front. Pharmacol.* 8:852. 10.3389/fphar.2017.00852 29213238 PMC5702848

