# Supplementary material for: Discovery of a Novel Nav1.7 Inhibitor From Cyriopagopus albostriatus Venom With Potent Analgesic Efficacy
Source: Front Pharmacol. 2018 Oct 16;9:1158. doi: 10.3389/fphar.2018.01158 (PMC6198068; doi:10.3389/fphar.2018.01158)
Supplement: Supplementary file 1 [file Table_1.DOCX]

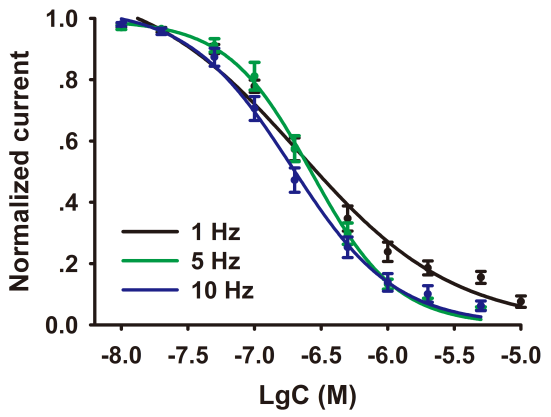


Figure S1 Use/frequency dependence of Ca2a inhibition for Na_v_1.7 channel (n = 4-5).


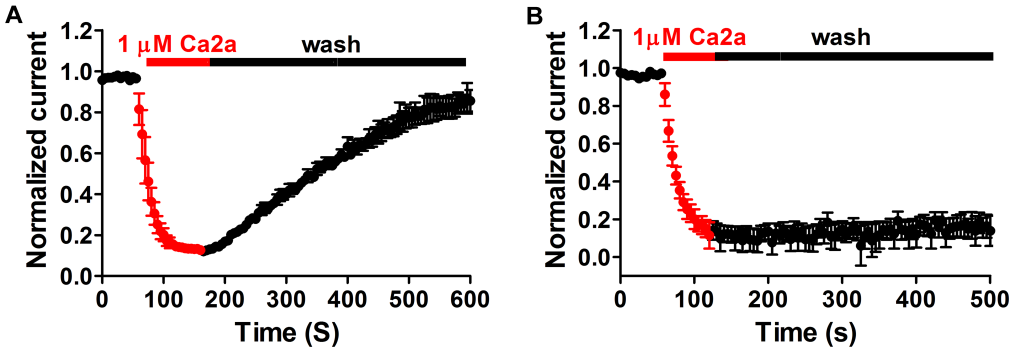


Figure S2 Inhibition of wild type and mutant channels by 1 µM Ca2a and recovery upon washing with bath solution plotted as normalized current. (A) Time course of Na_v_1.7 inhibition by 1 µM Ca2a and recovery upon washing, τ_on_ and τ_off_ are 18.0 ± 2.5 s and 295.6 ± 27.5 s, respectively (n = 4). (B) Time course of Na_v_1.7/D816N inhibition by 1 µM Ca2a and recovery upon washing, τ_on_  is 18.4 ± 1.3 s (n = 4).


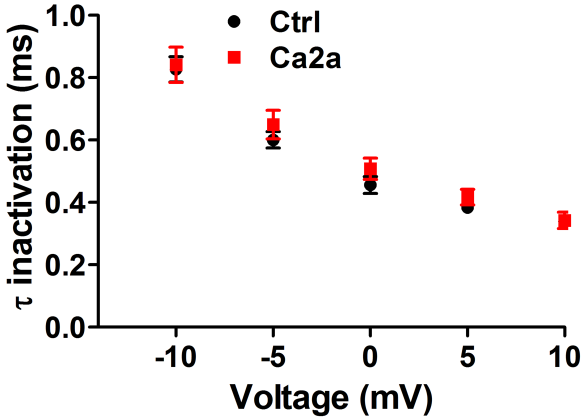


Figure S3 Fast inactivation kinetics in Na_v_1.7/D816N channel. 0.2 µM Ca2a did not alter inactivation time constant of Na_v_1.7/D816N between -10 mV and +10 mV (n = 7).
